# Focus on environmental footprint tools for sustainability: an overview of contributions

**DOI:** 10.1088/1748-9326/addb64

**Published:** 2025-06-06

**Authors:** James N Galloway, Jana E Compton, Allison M Leach

**Affiliations:** 1 Department of Environmental Sciences, University of Virginia, Charlottesville, VA, United States of America; 2 US EPA, Office of Research and Development, Corvallis, OR, United States of America; 3 Sustainability Institute, University of New Hampshire, Durham, NH, United States of America

Environmental footprint estimation aims to address the challenge of using resources efficiently for food and energy production by revealing effective approaches to minimize the negative consequences to human and ecosystem health. Therefore, footprint tools are particularly well-situated for making connections to consumers, which is important because sustainability goals are often dependent on changing consumer behavior.

Since the first papers in the early 21st century, the nitrogen (N) footprint concept has been applied to individuals, institutions, communities, and countries. The role of this special issue focus collection of papers is to bring together new findings from related footprint efforts, along with those already developed at the country, campus, and community scale. This focus collection also explores links with other environmental footprints and how applying these footprint tools can help meet community and campus sustainability goals.

The twenty-seven papers in this collection (table 1), published from 2019–2025, illustrate that there has been great progress in the science of calculating footprints of human activities on water use, land use, and element release to the environment. The paper topics can be broadly grouped into the categories: general, agricultural/food, institutional, country/individual, elemental and community. The study locations span four continents, with eight papers from Asia (China, Japan and Palau), seven from Europe (Belgium, Denmark, Germany, Spain, Sweden and Ukraine), and eight from North America (Canada and the United States). Four papers took a global perspective, including two papers that employed a social science perspective. The scales of the footprint calculators also varied: cities, states/-provinces or counties (6), country (15), individual (8), and institutional level (4). Twenty-two of the papers examined N footprints, with seven of these adding carbon (C) (3), water (2), greenhouse gas (1) or phosphorus (P) (3) footprints. Three papers used other approaches to examine environmental, land or ecological footprints.

Many of the publications provide improved methods for estimating footprints of individuals. If the consumer (person, community, institution) is at the end of the N footprint chain, then food/agriculture is arguably at the beginning. In this category, we have the following contributions:

- Hayashi *et al* (2020) provide the methodology to expand the N footprint concept to protein-free foods, such as oils and sugars which in some cases can be an important component of consumed food.
- Wang *et al* (2020) discuss N hotspots in Chinese agricultural systems by combining two approaches to N loss in cropping systems—the DeNitrification—DeComposition (DNDC) model which tracks specific N fluxes and the reactive nitrogen spatial intensity (NrSI) approach which calculates N footprints on a per-area basis. This integrated method is useful to identify areas and/or cropping systems with particularly high Nr losses.
- Shindo *et al* (2021) use a top-down method to look at N footprints in China and Japan. They found that the N footprint in China is larger than Japan by about ~15% due to greater protein consumption. They also found that when the top-down method was compared to the bottom-up N footprint method, the former was larger by ~10% due to the consumption of oils and sugars.
- Cattell Noll *et al* (2020) contrast the N footprint of organic vs. conventionally produced food in the US. They found that the N footprint of organic food production is not better than conventional, and is sometimes worse (e.g., beef). This is because pasture-raised beef takes longer to come to slaughter weight and thus, more N is processed by the animal.
- Mori *et al* (2020) examine the N lost to the environment by several Japanese milk and beef production systems to help consumers select environmentally sustainable animal protein products.
- Mahjabin *et al* (2021) explore virtual N and water transfers in US food trade networks. They found that most of the virtual N transfers move from states with high environmental impairments to states with lower rates of impairments. These findings provide a needed enhancement of the footprint approach that can have value for eutrophication and water use management in the US.
- Aguilera *et al* (2021) document the long-term trajectories of the C footprint due to N fertilization in Spanish agriculture. They found that significant energy efficiency gains of industrial fertilizer production were largely offset by the changes in the fertilizer mix. Downstream nitrous oxide emissions associated with ammonia volatilization and nitrate leaching increased tenfold. Their results suggest that mitigation efforts should not only aim to increase N use efficiency but also consider water management, fertilizer type, and fertilizer manufacture as key drivers of emissions.
- Oita *et al* (2021) uses the nutrient-extended input–output method to determine the nutrient footprint as a sum of direct and indirect inputs throughout the supply chains from different sources of nutrients. They found that this method provides quantitative insights for all stakeholders of food consumption and production to improve the nutrient use efficiencies of agro-food supply chains.
- Einarsson *et al* (2022) address the contribution of food waste to the Swedish personal N footprint. They conclude that ‘76% of food waste N is unavoidable (bones and other parts not commonly eaten). Avoidable food waste is about 7% of the edible food supply, implying that a hypothetical complete elimination of food waste would reduce emissions by about 7%’.
- Vos *et al* (2025) address the N footprint of Denmark with an eye towards balancing environmental regulation vs. individual environmental responsibility. The three key abatement strategies are shifting from meat to plant-based alternatives, improving nitrogen use efficiency and reducing waste and enhancing recycling throughout the entire farm to plate supply chain.

Personal N footprint calculators have been developed for *~*20 countries, and the two most recent ones are highlighted in this focus collection. Klement *et al* (2021) present the N footprint tool for Germany, using a revised version of the original tool. In addition, they proposed improvements to the tool to increase its informative value and user acceptance of an N-Calculator. McCourt and MacDonald (2021) showcase a footprint calculator for Canada, and they have gone a step beyond the whole-nation approach by calculating the N footprints for each of Canada’s 10 provinces. They found that most variation of the per-capita footprint across provinces is due to the fossil fuels sector, including emissions from energy generation and the oil and gas industry. Their study emphasizes the need to consider how heterogeneous geographic contexts contribute to national N footprints.

Moving from the N footprints of individuals in different countries to N footprints of people living in communities, we examine two types of scales. The first is at the community scale. Generally, the methodology is the same for a person living in a country as for a person living in a community, but there may be implications for food, transport and energy because of commonalities at this scale.

- Nakamura *et al* (2021) examine the land use of diets from the perspective of a Pacific Ocean Island. They found that the dietary lifestyles of communities depend on external land use through the global supply chain, showing that the dietary habits of the community are heavily dependent on processed and imported foods.
- Xian *et al* (2022) investigate the N footprints of China’s urban regions over a 20 year period to understand the challenges of advancing sustainable development on a regional basis. They demonstrated the potential of the N footprint tool for the assessment of sustainable development in urban agglomeration, which may prove instructive for broader research on sustainable Nr management.
- He *et al* (2024) address the contradiction between resource supply and demand has brought ecological security. They recommend that ‘it is advised to boost investment in eco-friendly tech, foster green economy growth, and prioritize renewable energy development’.
- Gomez and Grady (2023) explore the interplay between food supply resilience and environmental impacts in US cities. They provide ‘insights into potential trade-offs and opportunities for creating sustainable urban food systems in the US, underscoring the need for strategies that consider both resilience and environmental implications’.

The second scale is the institutional scale, namely academic institutions. This does not address the N footprint of individuals, but rather of collections of people in institutions. There are three instances:

- Arsenault *et al* (2019) examine the contribution of travel to the N and C footprints of the University of Montréal, Canada. They addressed the main reasons for traveling, the most frequent destinations, and the factors preventing researchers from reducing their travel-related environmental impact.
- MacDonald *et al* (2020), using data from McGill University and University of Montréal, Canada, examined the geographic vs institutional drivers within these urban universities. They found that a shared geographic context of a dense city with plentiful off-campus housing, food options, and access to hydroelectricity shapes the absolute N footprints of Montréal’s two main universities more than the divergent institutional characteristics that influence their relative N footprints.
- Messenger *et al* (2021) use the Marine Biological Laboratory in Woods Hole, MA, USA, to examine the effectiveness of low-effort N footprint reductions in small institutions. They found that significant reductions can occur with minimal institutional investments.

In particular, this special issue presents several papers that provide some of the first estimates of anthropogenic P footprints (Metson *et al* 2020, Papangelou *et al* 2021), and comparisons of the N and P footprints of food (Metson *et al* 2020). Food production has been a focus of many of these papers, and we are able to see the wide range of differences in per capita and per country inputs based upon the variations in diet, food sourcing, and the transportation of food.

- Metson *et al* (2020) examine the US consumer P footprint from the perspective of where N and P diverge. They found that P is managed less efficiently than N in food production systems but more efficiently removed from wastewater. Moreover, they also found that while strategies like reducing meat consumption will effectively reduce both N and P footprints by decreasing overall synthetic fertilizer nutrient demands, consideration of how food production and waste treatment differentially affect N and P releases to the environment inform eutrophication management.
- Papangelou *et al* (2021) examine the P footprint for urban diets. Specifically, they determined the P footprint for the food consumption in Brussels and used it to compare different strategies towards increased circularity: waste reuse, waste reduction, dietary changes and shifts to locally produced food. They found that P footprints could be lowered by dietary changes but would increase if production was limited to Belgium.

Two papers explicitly examined islands and their unique challenges to reduce environmental footprints associated with food produced within the island or imported from other areas (Nakamura *et al* 2021, Hamada *et al* 2023). Internal sourcing of manure from within the island was seen as a way to reduce the N footprint associated with imported food and fertilizer on Ishigaki Island, Japan (Hamada *et al* 2023). In Palau, reducing external food supply and improving the internal food production was viewed as a way to reduce the land footprints associated with food (Nakamura *et al* 2021).

One interesting and timely paper identified the role that displacement of people in the war in Ukraine impacts the release of N to the environment (Medinets *et al* 2024). People were moved into areas with poor infrastructure, leading to increased N losses that may have broader environmental impacts.

A global paper by Malik *et al* (2022) examines the drivers of global N emissions at a detailed country level and break down the change in emissions into contributions from domestic footprints and a rest-of-world footprint. They highlight that food production coupled with growing international trade is increasing Nr emissions worldwide.

Two papers used a social science perspective to understand how people learn and apply the information from different types of footprint calculators (Collins *et al* 2020, Sörqvist *et al* 2020). Both papers indicated that more information is needed to communicate the paths for people to reduce their footprints when given a variety of choices.

- Collins *et al* (2020) used the Global Footprint Network’s personal Footprint calculator, to understand the profile of calculator users and assess the contribution of calculators to increasing individual awareness and encouraging sustainable choices. They found that only 23% of users indicated the calculator provided them with the necessary information to make actual changes to their life and reduce their personal Footprint. Similar to other calculators, this tool has limited utility for enabling individuals to convert new knowledge into action.
- Sörqvist *et al* (2020) address an incredibly important topic, the psychological obstacles to the use of footprint tools, specifically carbon (C). They concluded that environmental footprint tools might connect consumer choices with environmental consequences accurately. However, people struggle to evaluate the environmental consequences of bundles of items when informed that these items vary in their C footprint. They suggest that one area for future research is to find more effective ways to communicate to consumers how to sum multiple C footprints.

## 1. Summary

The first quarter of the 21st century has seen a rapid growth in the number and type of footprint tools. While initially they were focused on individuals, now they are available for institutions and communities. And the broader perspectives from social science reveal and reinforce the importance of the need for multidisciplinary approaches to strengthen the communication and use of information around environmental footprints.

As reflected in Galloway *et al* (2024) in this special issue, as the science of footprints is building tools and developing quantification frameworks, the current challenge is to get the tools and information into the hands of people, institutions, and other decision-makers to use them. Information transfer entails working together to prioritize and develop communication pathways for better managing environmental footprints.

## Figures and Tables

**Table 1.** List of all papers, organized by footprint type including title, citation, location of the work, scale, footprint type, primary focus, and conclusion of each paper.

Title	Citation	Location	Scale	Footprint type	Primary focus	Conclusion
Long-term trajectories of the C footprint of N fertilization in Mediterranean agriculture (Spain, 1860–2018)	Aguilera *et al* 2021 *Environ. Res. Lett*. 16 085010	Spain	Country	C, N	Identification of emission hotspots, accounting for C footprint of fertilizers, fertilizer composition shifts over time	Emission factors should be developed for different regions, increased production was offset by increase in emissions
Psychological obstacles to the efficacy of environmental footprint tools	Sörqvist *et al* 2020 *Environ. Res. Lett*. 15 091001	Europe but perhaps global	Individual	C, N	Understanding how people view and make decisions related to their C footprint	People have a difficulty summarizing impacts; suggestion is to arrange the options so that people are steered toward environmentally positive behaviors
The environmental footprint of academic and student mobility in a large research-oriented university	Arsenault *et al* 2019 *Environ. Res. Lett*. 14 095001	Canada	Institution	C, N	Transportation	Air travel emissions were the largest part of the transportation footprint
Spatiotemporal changes and drivers of ecological security based on an improved ecological footprint model: the case of Hubei Province, China	He *et al* 2024 *Environ. Res. Lett*. 19 064049	Hubei Province China	Province	Ecological footprint	Improved ecological footprint modeling	Substantial increases in footprints from 2000–2020, recommendations to improve
Living within a One Planet reality: the contribution of personal Footprint calculators	Collins *et al* 2020 *Environ. Res. Lett*. 15 025008	Global	Individual	Environmental	Survey of footprint calculator use by individuals	Footprint calculators were seen as valuable for awareness but lacking in information to allow respondents to take action in their lives.
Global land use of diets in a small island community: a case study of Palau in the Pacific	Nakamura *et al* 2021 *Environ. Res. Lett*. 16 065016	Ollei Village, Republic of Palau	Country	Land footprint from food	Quantified N inputs from imported foods using food ingredient surveys	Large increase in external food supply affecting community and environment
Assessment of nitrogen hotspots induced by cropping systems in the Bohai Rim region in China by integrating DNDC modeling and the reactive nitrogen spatial intensity (NrSI) framework	Wang *et al* 2020 *Environ. Res. Lett*. 15 105 008	Bohai Rim Region, China	Counties	N	Spatial hotspots of N release using the Nr Spatial Intensity framework	NH_3_ volatilization and nitrate leaching were 24% and 19% of Nr inputs, respectively. Identification of hotspots could allow prioritization of areas for mitigation.
Drivers of global nitrogen emissions	Malik *et al* 2022 *Environ. Res. Lett*. 17 015006	Global	Countries	N	Trade-based N emissions, incorporates affluence & population	Efficiency of N use to produce products has increased, but more products are being produced overall, increasing the footprint. Food production and trade are driving this increase.
Comparison of food supply system in China and Japan based on food nitrogen footprints estimated by a top-down method	Shindo *et al* 2021 *Environ. Res. Lett*. 16 045003	China and Japan	Country	N	Country comparison, changes over time	China’s larger per capita footprint linked to higher protein intake, identifies missing food products that have important effects on food footprint
Nutrient-extended input-output (NutrIO) method for the food nitrogen footprint	Oita *et al* 2021 *Environ. Res. Lett*. 16 115 010	Japan	Country	N	Using nutrient-extended input-output modeling to estimate country-level footprint, focusing on food	A standardized I-O approach provides an improved estimate for Japan and may be useful as a model for other countries to standardize for global analyses
The nitrogen footprint of organic food in the United States	Cattell Noll *et al* 2020 *Environ. Res. Lett*. 15 045004	US	Country	N	Organic food production footprint	The overall N footprint of most organic foods is similar to conventional foods, except for beef which is much higher. Organic production has lower inputs of new N, except for beef.
Provincial nitrogen footprints highlight variability in drivers of reactive nitrogen emissions in Canada	McCourt and MacDonald 2021 *Environ. Res. Lett*. 16 095007	Canada	Country, Province, Individual	N	Improved per capita footprints from agriculture, wastewater and fossil fuels, comparison of approaches	Fossil fuel sector drives differences between provinces, importance of exports
Calculation of a food consumption nitrogen footprint for Germany	Klement *et al* 2021 *Environ. Res. Lett*. 16 075005	Germany	Individual (and country-level)	N	Updated N calculator for Germany based on food virtual N factors	Compared different approaches to find reasonable agreement
Nitrogen loss to the environment due to various nitrogen-use efficiencies during milk and beef production in Japan	Mori *et al* 2020 *Environ. Res. Lett*. 15 125 007	Japan	Individual (and country-level)	N	Updated Virtual N Factors and NUE for food	Found a range of VNFs and NUE for different types of milk and beef production; understanding the driving factors could improve product sustainability and consumer choices
The nitrogen footprint of Ukraine: why personal consumption matters	Medinets *et al* 2024 *Environ. Res. Lett*. 19 024023	Ukraine	Individual (and country-level)	N	Determining and improving individual N footprint (NF) calculations, comparing with other countries, suggestions for reducing NFs	Displacement of people to areas with poor infrastructure led to increased N losses
The Danish nitrogen footprint: balancing regulation with individual environmental responsibility	Vos *et al* 2025 *Environ. Res. Lett*. 20 054026	Denmark	Individual (and country-level)	N	Addresses the N footprint of Denmark with an eye towards balancing environmental regulation vs. individual environmental responsibility.	The three key abatement strategies: shift from meat to plant-based alternatives, improve nitrogen use efficiency and reduce waste and enhancing recycling throughout the entire farm to plate supply chain.
Geographic versus institutional drivers of nitrogen footprints: a comparison of two urban universities	MacDonald *et al* 2020 *Environ. Res. Lett*. 15 045008	Canada	Institution	N	Spatial patterns	Comparison of universities found some campus specific factors were important, but the geographic context was a more important driver of the N footprint
Assessing nitrogen flow and nitrogen footprint in the food system of a subtropical island with a scenario to mitigate nitrogen load impacted by trade-dependent agriculture	Hamada *et al* 2023 *Environ. Res. Lett*. 18 075010	Ishigaki Island, Japan	Island	N	N footprint of island, focused on N inputs and food production	High dependence on food, feed and fertilizer imports, replacement of fertilizer with currently generated manure recommended to reduce N import and loss
The nitrogen footprints of China’s major urban agglomerations: understanding regional challenges to advance sustainable development	Xian *et al* 2022 *Environ. Res. Lett*. 17 045020	China	Large cities	N	Quantifying footprints in each city: food and energy footprints	High energy footprint in these urban agglomerations
The nitrogen footprint of Swedish food consumption	Einarsson *et al* 2022 *Environ. Res. Lett*. 17 104 030	Sweden	Individual (and country-level)	N	Improving food footprint estimates	Food waste is 7% of edible food supply, which seems quite low
Identifying and assessing effectiveness of alternative low-effort nitrogen footprint reductions in small research institutions	Messenger *et al* 2021 *Environ. Res. Lett*. 16 035014	US	Institution	N	Approaches to reduce emissions	Combining C and N footprint strategies had benefit of reducing the C footprint more than focusing on C alone
Footprint tools tiptoeing towards nitrogen sustainability	Galloway et al 2024 *Environ. Res. Lett*. 19 103 003	Global	Individual, institution, community	N, and one multi-element tool (N, C, P, water)	Review history, methods, and uptake of N footprints	Need for tools that are less complex and more complex
Concealed nitrogen footprint in protein-free foods: an empirical example using oil palm products	Hayashi *et al* 2020 *Environ. Res. Lett*. 15 035006	Global	Country	N	Calculating an N footprint for palm oil products	Improvement to N footprint by expanding the Virtual Nitrogen Free concept for crops that do not contain N.
The U.S. consumer phosphorus footprint: where do nitrogen and phosphorus diverge?	Metson *et al* 2020 *Environ. Res. Lett*. 15 105 022	US	Country	N, P	Food production	Comparing N and P found that P is less efficiently managed than N in food systems but more efficiently managed in wastewater.
Virtual nitrogen and virtual water transfers embedded in food trade networks across the US	Mahjabin *et al* 2021 *Environ. Res. Lett*. 16 045015	US	States	N, water	Comparing food footprints by state	Virtual N transfers move from states with high impairments to states with lower impairments
A balancing act: the interplay of food supply chain resilience and environmental sustainability in American cities	Gomez and Grady 2023 *Environ. Res. Lett*. 18 124 022	US	Cities	N, water and GHG emissions	Using county-level agricultural data to study GHG emissions, and N losses of urban food systems across the US	Wide range of food supply chain resilience across US cities, potential to improve sustainable food systems
A resource-based phosphorus footprint for urban diets	Papangelou *et al* 2021 *Environ. Res. Lett*. 16 075002	Brussels, Belgium	City	P	Improving P footprint approach for urban area, scenario modeling	Diet shifts away from livestock can reduce the footprint and increase circular food systems in cities
